# Supplementary material for: Changes in Brain Activation through Cognitive-Behavioral Therapy with Exposure to Virtual Reality: A Neuroimaging Study of Specific Phobia
Source: J Clin Med. 2021 Aug 9;10(16):3505. doi: 10.3390/jcm10163505 (PMC8397119; doi:10.3390/jcm10163505)
Supplement: Supplementary file 1 [file jcm-10-03505-s001.zip › jcm-1292634-supplementary.pdf]

S1 Table. Functional brain activation

Activation	Active control group (real images)									Intervention group (virtual images)								
	Pre-treatment			Post-treatment			Pre > Post			Pre-treatment			Post-treatment			Pre > Post		
	p	$F_{(1,58)}$	*k*	*p*	$F_{(1,58)}$	*k*	*p*	$t_{(58)}$	*k*	*p*	$F_{(1,58)}$	*k*	*p*	$F_{(1,58)}$	*k*	*p*	$t_{(58)}$	*k*
**Thalamus**																		
Right Hemisphere	.000*	53.56	85	.000*	44.50	21	.000*	5.03	57	.000*	25.76	45	.000 *	3.79	9	.000*	3.50	7
Left Hemisphere	.000*	58.48	61	.000*	45.90	16	.000*	4.75	27	.000*	23.95	27	-	-	-	-	-	-
**Amygdala**																		
Right Hemisphere	.000*	31.71	15	-	-	-	.003	2.82	3	.000*	23.76	11	-	-	-	.001	3.43	5
Left Hemisphere	.000*	29.56	11	.000*	16.08	5	-	-	-	.000*	24.96	10	-	-	-	.000*	3.60	5
**Occipital cortex**																		
Right Hemisphere	-	-	-	-	-	-	.003	2.86	3	-	-	-	-	-	-	-	-	-
Left Hemisphere	-	-	-	-	-	-	.000*	3.52	5	-	-	-	-	-	-	.001	3.28	3
**Frontal orbital cortex**																		
Right Hemisphere	.000*	30.56	5	-	-	-	.001	3.37	5	.000*	26.20	4	-	-	-	.000*	3.59	4
Left Hemisphere	.000*	24.77	4	-	-	-	-	-	-	.000*	22.41	8	-	-	-	.000*	3.97	4
**DPC**																		
Right Hemisphere	.000*	62.39	31	.000*	30.48	10	.000*	3.95	6	.000*	40.78	21	-	-	-	.000*	4.00	6
Left Hemisphere	.001	62.24	20	.000*	2.47	4	.000*	4.43	6	-	-	-	-	-	-	-	-	-
**VPC**																		
Right Hemisphere	.000*	17.07	3	-	-	-	.000*	4.53	3	.001	12.94	8	-	-	-	-	-	-
Left Hemisphere	-	-	-	-	-	-	-	-	-	-	-	-	-	-	-	.000*	3.83	9
**ACC**																		
Right Hemisphere	-	-	-	-	-	-	.000*	3.54	4	-	-	-	.001	13.04	3	-	-	-
Left Hemisphere	-	-	-	-	-	-	-	-	-	-	-	-	-	-	-	.001	3.39	17
**Insula**																		
Right Hemisphere	.000*	31.32	35	-	-	-	.001	3.18	13	.000*	32.48	56	.000*	15.72	7	.000*	4.56	23
Left Hemisphere	.000*	17.13	10	-	-	-	.001	3.16	8	.000*	31.88	55	-	-	-	.000*	5.49	48
**Hippocampus**																		
Right Hemisphere	.001	81.45	23	.008	57.46	12	-	-	-	.000*	57.20	30	-	-	-	-	-	-
Left Hemisphere	.001	71.82	22	.003	61.18	16	-	-	-	-	-	-	-	-	-	-	-	-
**Fusiform gyrus**																		
Right Hemisphere	.000	147.78	126	.000*	102.9	95	.000*	4.47	7	.000*	60.40	40	.000*	57.20	30	.000*	3.76	7
Left Hemisphere	.000	122.87	127	.000*	108.9	104	.000*	4.50	11	.000*	77.42	56	.000*	84.02	38	.000*	3.41	3

Activation	Active control group (real images)												Intervention group (virtual images)											
	Pre-treatment			Post-treatment			Pre > Post			Post > Pre			Pre-treatment			Post-treatment			Pre > Post			Post > Pre		
	p	$F_{(1,58)}$	*k*	*p*	$F_{(1,58)}$	*k*	*p*	$t_{(58)}$	*k*	*p*	$t_{(58)}$	*k*	*p*	$F_{(1,58)}$	*k*	*p*	$F_{(1,58)}$	*k*	*p*	$t_{(58)}$	*k*	*p*	$t_{(58)}$	*k*
Precuneous																								
RH	-	-	-	-	-	-	-	-	-	.000*	3.90	6	-	-	-	-	-	-	.000*	4.21	18			
LH	-	-	-	-	-	-	.000*	3.59	3	.000*	4.17	9	-	-	-	-	-	-	-	-	-	.000*	3.90	21

S1 Table legend
* $p < .001$

ACC: Anterior cingulate cortex; DPC: Dorsolateral prefrontal cortex; LH: Left Hemisphere; RH: Right Hemisphere; VPC: Ventromedial prefrontal cortex
